# Adaptive responses of outer membrane porin balance of *Yersinia ruckeri* under different incubation temperature, osmolarity, and oxygen availability

**DOI:** 10.1002/mbo3.354

**Published:** 2016-04-01

**Authors:** Evgeniya Bystritskaya, Anna Stenkova, Dmitriy Chistuylin, Nadezhda Chernysheva, Valentina Khomenko, Stanislav Anastyuk, Olga Novikova, Alexander Rakin, Marina Isaeva

**Affiliations:** ^1^ G.B. Elyakov Pacific Institute of Bioorganic Chemistry FEB RAS690022 Prospect 100‐let Vladivostoku 159VladivostokRussia; ^2^ The School of BiomedicineFar Eastern Federal University690950 Suchanova str. 8VladivostokRussia; ^3^ Max von Pettenkofer Institute for Hygiene and Clinical Microbiology of Ludwig Maximilians‐University80336 Pettenkofer str. 9aMunichGermany

**Keywords:** Bacteria, environmental signal/stress responses, gene expression/regulation, outer membrane proteins, porins.

## Abstract

The capability of *Yersinia ruckeri* to survive in the aquatic systems reflects its adaptation (most importantly through the alteration of membrane permeability) to the unfavorable environments. The nonspecific porins are a key factor contributing to the permeability. Here we studied the influence of the stimuli, such as temperature, osmolarity, and oxygen availability on regulation of *Y. ruckeri* porins. Using qRT‐PCR and SDS‐PAGE methods we found that major porins are tightly controlled by temperature. Hyperosmosis did not repress OmpF production. The limitation of oxygen availability led to decreased expression of both major porins and increased transcription of the minor porin OmpY. Regulation of the porin balance in *Y. ruckeri*, in spite of some similarities, diverges from that system in *Escherichia coli*. The changes in porin regulation can be adapted in *Y. ruckeri* in a species‐specific manner determined by its aquatic habitats.

## Introduction

*Yersinia ruckeri* is a Gram‐negative bacterium intermittent between planktonic and host interaction states (Austin and Austin 1987). It causes systemic infection (known as enteric redmouth disease) in fish, mostly in the family Salmonidae. The disease is characterized by hemorrhagic septicemia and high fish mortality. Significant economic losses in the aquaculture industry are associated with yersiniosis outbreaks, which generally occur under poor sanitary or severe stress conditions (Furones et al. 1993; Tobback et al. 2007). *Yersinia ruckeri* is able to survive for prolonged period in fresh and salt water after an outbreak of yersiniosis (Thorsen et al. 1992). Wild and clinically healthy fish can be *Y. ruckeri* carriers providing transmission of the bacterium to other fishes (Willumsen 1989).

Obviously, the capability of *Y. ruckeri* to survive in the aquatic systems reflects adaptation of the organism to the disadvantageous environment (low temperature, low nutrients, high/low osmolarity, oxygen deficit, antibiotics). There are different surviving strategies developed during bacterial evolution, one of them is alteration of outer membrane permeability. General porins, the most abundant outer membrane proteins (OMPs), are responsible for the permeability of the membrane to small hydrophilic polar molecules. The balance of major OMPs, known as OmpF and OmpC, is achieved through its complex regulation often reciprocal in response to many environmental factors, such as temperature, osmotic pressure, and pH (Liu and Ferenci 2001). Alteration in the porin balance has been observed for a number of clinical isolates making contribution to the adaptive bacterial response to the treatment (Pagès et al. 2008; Bystritskaya et al. 2014). In addition to major general porins, there are minor ones expressed at very low levels (Prilipov et al. 1998). Potentially, some of them contribute to the bacterial adaptation to balance the expression levels of the major OMPs. Previously, we characterized new minor porin, named OmpY, from *Y. pseudotuberculosis* (Solo'veva et al. 2011). The protein demonstrates features of classical nonspecific porins similar to OmpF and OmpC. The environmental conditions of expression of this porin in the cells are poorly understood and little is known about its function.

Recently, we defined two general porins, OmpF and OmpC, in *Y. ruckeri* outer membrane (Chistyulin et al. 2012). However, little is known about influence of environmental clues on the synthesis of nonspecific porins in *Y. ruckeri*. In the present work we have used real‐time PCR and SDS‐PAGE methods to investigate the effect of three different environmental conditions, namely temperature, osmolarity, and oxygen availability, on the expression pattern of *Y. ruckeri* porins. Studies of environmental factors that control the regulation of porins can provide insight into potential regulatory relationships among these genes, and help in further investigations of adaptive responses to environmental conditions and their effect on bacterial tolerance and pathogenicity. This applies especially to the aquatic environments where both the host and the bacterium are greatly influenced by the fluctuations of ambient water conditions.

## Materials and Methods

### Strain and growth conditions

Strain *Y. ruckeri* KM 821 was grown overnight in LB medium at 26°C with aeration. The overnight culture was diluted 1:50 into fresh LB medium to 200 mL, changing the following conditions: NaCl concentration (0, 150, 300 mmol/L), temperature (8, 26, 37°C), aeration (200 rpm), and limited oxygen availability, and incubated to mid‐exponential growth phase (OD_600 _= 0.4–0.6). Then 1 mL of the culture was centrifuged, and the cell pellet was placed in the RNA Protect bacterial reagent (Qiagen, Moscow, Russia) to stabilize total RNA. The rest culture was sedimented by centrifugation and 150 mg of the wet bacterial pallet was used for porin extraction.

### Gene expression measurement by qRT‐PCR

Total RNA was extracted from two biological replicates using the Aurum Total RNA Mini Kit (BioRad, Moscow, Russia) comprising a treatment with RNase‐free DNase I to eliminate DNA contamination. First‐strand cDNA synthesis was performed from 2 *μ*g of total RNA using the MMLV RT kit (Evrogen, Moscow, Russia) and random primer according to the manufacturer's instruction.

The cDNAs were subsequently quantified by real‐time PCR amplification with primers specific to the outer membrane protein genes and 16S rDNA gene (Table 1), GoTaq DNA polymerase (Promega) and dye EvaGreen (Biotium, California, USA) on the iCycler IQ5 (BioRad). The following PCR cycles were used: 2 min at 95°C (95°C for 20 sec, 55°C for 20 sec, 72°C for 20 sec) × 45, and recording of a melting curve. The absence of nonspecific product amplification was checked by melting curve analysis. Each run included negative controls. Expression level results were standardized relative to the transcription level of the housekeeping gene 16S rDNA for each isolate. The relative change in the *ompF*,* ompC*,* ompY* expression was calculated as the ratio of reference to target using the ΔΔCt method, where Ct is the cycle threshold.

**Table 1:** Primers for qRT‐PCR analysis of porin gene expression in *Yersinia ruckeri*

Gene	Forward primer	Reverse primer
*ompF*	5′ ACCGCAACACCAACTTCTTC 3′	5′ ACCGTCGCCATTCTCATCTT 3′
*ompC*	5′ TGCTGACTTTGGTTCTCTGGA 3′	5′ GTTGCCATTTTTGCCCTGAT 3′
*ompY*	5′ ATGTTCCCTGAGTTCGGTGGCG 3′	5′ AACAGACAGGCCGTAACCGTCG 3′
16S rDNA	5′ TTTGTTGCCAGCACGTAATGGT 3′	5′ GCGAGTTCGCTTCACTTTGTATCT 3′

Real‐time PCR was carried out on two independent biological replicates each containing three technical replicates. The results are presented as the mean ± SD.

Statistical analysis was performed with software STATISTICA version 10 (StatSoft inc., Oklahoma, USA) (StatSoft inc., Oklahoma, USA) (StatSoft inc., Oklahoma, USA) using Student's *t* test. *P* < 0.05 was considered statistically significant.

### Protein isolation and identification

Porins were isolated and purified as described previously (Chistyulin et al. 2012). Each microbial biomass sample was treated with 0.5% sodium sarcosylate solution for 12 h and then centrifuged. The precipitate was suspended in 30 mmol/L Tris‐HCl (buffer A, pH 7.8) containing 2% SDS and centrifuged for 1 h at 25,000 *g*. The complex of peptidoglycan (PG) and OM proteins in the precipitate was treated with DNAse (Fermentas). The fraction containing PG‐associated proteins was extracted with buffer A containing 1% SDS and 0.5 mol/L NaCl. Protein fractions were analyzed by SDS‐PAGE. Gels were stained with Coomassie brilliant blue G250 in 3.5% perchloric acid solution. Original SDS‐PAGE are presented in Figures S1 and S2.

Identification of proteins in sample, obtained at 26°C, 300 mmol/L NaCl with aeration, was performed by MALDI‐TOF mass spectrometric analysis. Mass spectra were recorded using an Ultraflex III TOF/TOF mass spectrometer (Bruker Daltonics) equipped with a high mass HMD 1 detector (CovalX AG) in linear positive ion detection mode. A saturated solution of 3,5‐dimethoxy‐4‐hydrocinnamic (sinapinic) acid (10 mg/mL) in an acetonitrile–0.1% TFA mixture (70% acetonitrile) was used as the matrix. The sample was mixed with the matrix and spotted onto a target by the dried droplet method. 0.05 mg/mL BSA was used to calibrate the mass spectrometer.

## Results and Discussion

### MALDI mass spectrometry of porins

**Figure 1:**
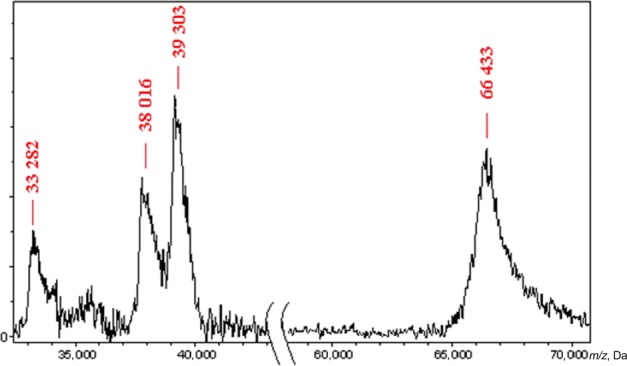
MALDI‐TOF mass spectrometry of proteins; 0.05 mg/mL BSA was used as a molecular weight marker.

To identify porins we performed an accurate measurement of their molecular masses by MALDI‐TOF mass spectrometry. For analysis, we used the protein sample obtained from *Y. ruckeri* bacterial culture grown at 26°C, 300 mmol/L NaCl with aeration. BSA was analyzed as a control. MALDI‐TOF data show the presence of two proteins (Fig. 1) with molecular weights corresponding to OmpF and OmpC ones calculated from sequence analysis (Table 2). Reasonable agreement between the measured and calculated masses was obtained for each protein.

**Table 2:** Comparison of molecular masses of *Yersinia ruckeri* porins measured by MALDI‐TOF mass spectrometry and calculated from their gene sequences

Protein	Molecular mass, Da		Difference, %
	Calculated	Measured	
OmpF	37,975	38,016	0.1
OmpC	39,341	39,303	0.1
OmpY	37,683	–	–
BSA			
Single protonated molecule	66,430	66,433	<0.01
Double protonated molecule	–	33,282	

### Effect of growth temperature

**Figure 2:**
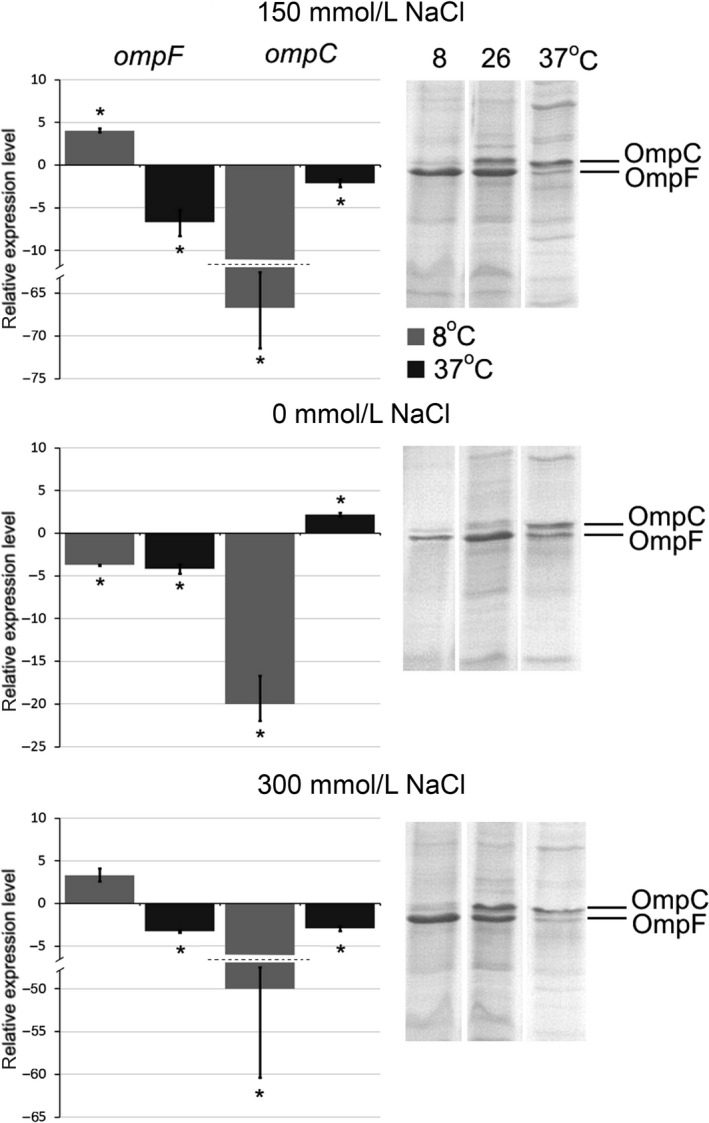
Effect of cultivation temperature on porin expression. Histograms display relative expression levels, which are reported as fold change compared to average expression level of reference groups. Temperature of 26°C was used as a reference. Columns represent the mean of triplicate measurements in two independent experiments; error bars represent the standard deviations. Asterisks above bars indicate significant differences compared to the reference (*P* < 0.05). On the right side are located 12% SDS‐PAGE of porin samples. Gel was stained with Coomassie blue. Lines were rearranged according to conditions.

We investigated the porin expression in *Y. ruckeri* at different temperatures (8°C, 26°C, and 37°C) similar to those it encounters in hosts or nature. *Yersinia ruckeri*, as all *Yersinia*, is a psychrophilic microorganism, it can grow and reproduce at cold temperatures from 4°C (the optimal growth temperature is 26–28°C). As shown in Figure 2, both qRT‐PCR and SDS‐PAGE data demonstrated that *Y*. *ruckeri* major porins are tightly controlled by temperature. In SDS‐PAGE, OmpF became predominant at low temperature (8°C), while OmpC dominated at higher temperature (37°C). However, both major porins were present in the outer membrane in almost equal amounts at the optimal growth temperature (26°C), excepting low osmolarity that resulted in considerable reduction in OmpC production.

According to the qRT‐PCR analysis, *ompF* transcription increased about four times at low temperature compared with 26°C. Increasing the temperature to 37°C promoted a 6.7‐fold reduction of *ompF* expression. Expression of *ompC* shows strong opposite effect at 8°C, it decreases up to 66 times. It should be noted that the maximum level of *ompC* transcription was observed at 26°C, and the further temperature increase to 37°C did not cause noticeable changes in the expression.

So, in case of *Y. ruckeri*, the low temperature dramatically reduces the transcription of *ompC* and induces *ompF*. Interesting to mention that a high temperature did not significantly affect the transcriptional level of *ompC* and OmpC remains the prevalent protein in the outer membrane because the *ompF* transcription decreases. Only a very few publication concern the modulation of porins in response to changes in temperature. For *Escherichia coli* the porin thermoregulation observed is reciprocal increase in OmpF and decrease in OmpC at 20°C and vice versa at 37°C (Lundrigan and Earhart 1984; Andersen et al. 1989). The same was found for *Serratia marcescens ompF* and *ompC* (Begic and Worobec 2006). However, these microorganisms are mesophylls and have higher optimal growth temperature (37°C) than *Y. ruckeri*. Gu et al. (2012) determined downregulation of OmpF *Y. enterocolitica* and constant production of OmpC at 37°C compared with 25°C. These data are consistent with our results. Constant *ompC* expression at 26 and 37°C maybe a specific feature of *Yersinia* or even of all psychrophilic members of Enterobacteriaceaea.

### Effect of media osmolarity

Osmolarity is an important environmental stimulus that affects the outer membrane permeability. Genes of the two major porins, OmpF and OmpC, belong to osmotically regulated genes that are under the control of the two‐component regulatory system, EnvZ‐OmpR (Matsubara and Mizuno 1999). The relative amounts of the *E. coli* OmpC and OmpF porins has been shown to vary reciprocally in response to medium osmolarity (Sleator and Hill 2002). OmpC becomes predominant porin at high osmolarity, whereas OmpF is prevalent at low osmolarity.

**Figure 3:**
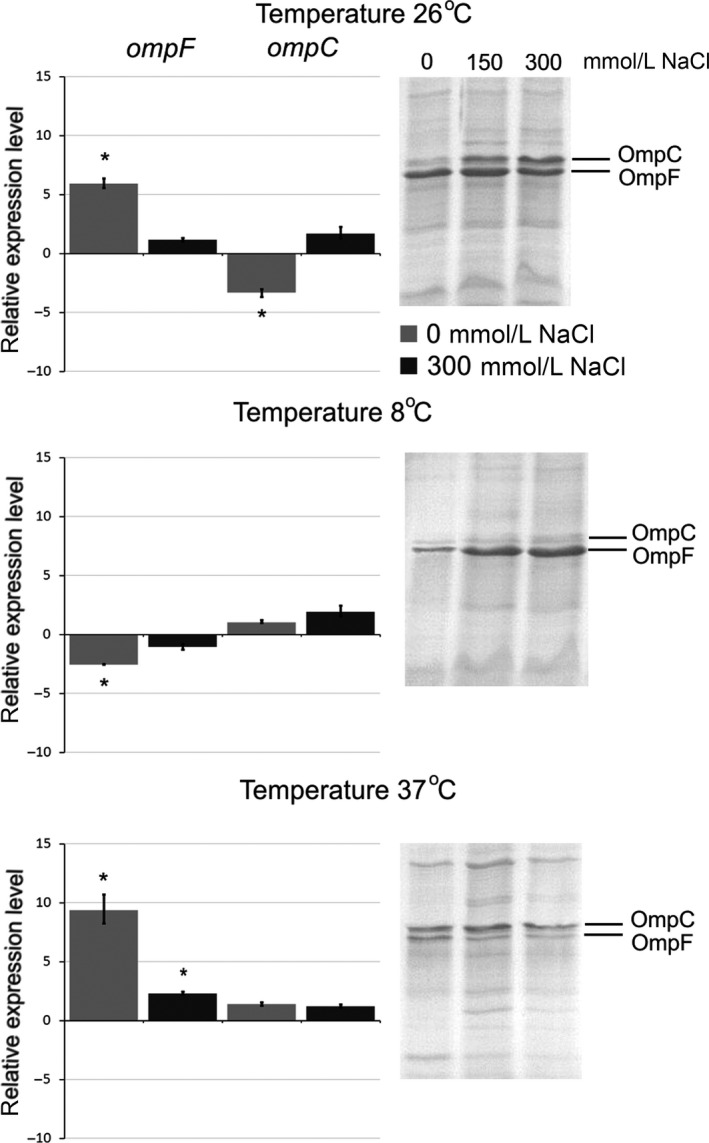
Effect of media osmolarity on porin expression. Histograms display relative expression levels, which are reported as fold change compared to average expression level of reference groups; 150 mmol/L NaCl condition was used as a reference. Columns represent the mean of triplicate measurements in two independent experiments; error bars represent the standard deviations. Asterisks above bars indicate significant differences compared to the reference (*P* < 0.05). On the right side are located 12% SDS‐PAGE of porin samples. Gel was stained with Coomassie blue. Lines were rearranged according to conditions.

We determined the effect of osmolarity on the transcription of *Y. ruckeri* porin genes. As shown in Figure 3, at optimal growth temperature (26°C) *ompC* transcription declined threefold as the osmolarity decreased to 0 mmol/L NaCl (hypo‐osmotic). However, *ompC* expression remained unchanged at 37°C and at 8°C both at hyperosmotic and hypo‐osmotic conditions. We suppose that no change in *ompC* expression under influence of different osmolarity, excluding hypo‐osmolar condition at 26°C, is due to the predominant effect of temperature. The transcriptional profile of *ompC* was also confirmed by SDS‐PAGE.

The transcription of *ompF* increased about 10‐fold at 37°C in hypo‐osmotic LB medium. The same effect was reproduced at 26°C, but the *ompF* transcription decreased by 2.5 times in hypo‐osmotic medium at 8°C (Fig. 3). In the protein level in hypo‐osmotic condition at 37°C OmpF rose, while the increasing of OmpF at 26°C was undetectable because the initial level of the protein at a given temperature was already high. In any case, OmpF of *Y. ruckeri* is either the main protein or its level is comparable to the level of OmpC in hypo‐osmotic conditions (37°C; 0 mmol/L NaCl). Interestingly, the hyperosmotic medium (300 mmol/L NaCl) did not decrease the *ompF* transcription and this strongly contrasts to what has been observed previously for *E. coli* (Alphen and Lugtenberg 1977). Consequently, the same effect of hyperosmolarity on *ompF* expression has been reported recently for another member of *Yersinia* genus, *Y. pestis* (Gao et al. 2011) and thus confirms our observations.

### Effect of oxygen availability

**Figure 4:**
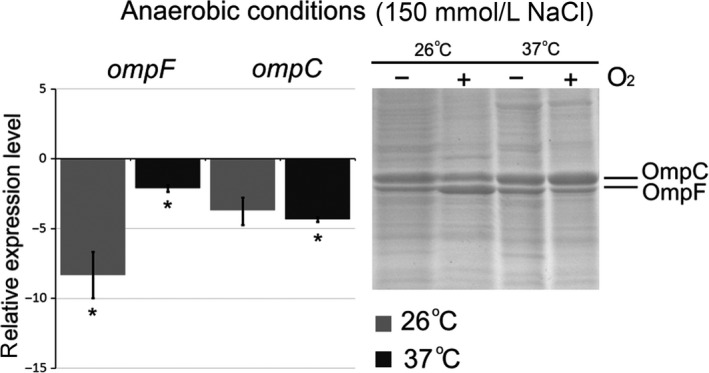
Effect of oxygen availability on porin expression. Histograms display relative expression levels, which are reported as fold change compared to average expression level of reference groups. The aerobic condition was used as a reference. Columns represent the mean of triplicate measurements in two independent experiments; error bars represent the standard deviations. Asterisks above bars indicate significant differences compared to the reference (*P* < 0.05). On the right side are located 12% SDS‐PAGE of porin samples. Gel was stained with Coomassie blue. Lines were rearranged according to conditions.

*Yersinia ruckeri* is a facultative anaerobe, which encounters anaerobic environments in its life cycle. Oxygen limitation is an important environmental factor that can also modulate OMP expression. To determine whether the porin expression in *Y. ruckeri* is regulated by oxygen, we cultivated the bacteria in aerobic or anaerobic conditions at various temperatures (26°C and 37°C) by normal osmolarity (150 mmol/L NaCl). The aerobic growth condition was achieved by vigorous agitation (200 rpm) in flasks. The anaerobic cultures were grown without agitation overlaid with sterile mineral oil. According to the OMP profiles in aerobic conditions (Fig. 4), OmpF was overproduced at 26°C compared with that at 37°C, while OmpC production increased at 37°C against 26°C. Under oxygen limitation at tested temperatures, no differences in the porin production were observed. Moreover, the oxygen deficit resulted in approximately eightfold reduction of *ompF* transcription at 26°C (Fig. 4) and about fourfold reduction of *ompC* transcription at 37°C.

Thus, the oxygen limitation decreased transcription of both *Y. ruckeri* major porins, with OmpC remaining the predominant porin in the outer membrane. It has been shown that in *E. coli*,* ompC* preferentially expressed in anaerobic conditions, whereas the *ompF* expression was favored in aerobic conditions (Ni et al. 1989).

### Participation of minor OmpY porin in environmental adaptation

Our understanding of porin balance regulation in response to the environmental clues should not be restricted only to the studying of the major porins. The existence of minor (quiescent) porins should be taken into account to achieve a comprehensive picture of the bacterial adaptation mechanisms. To date, our knowledge of the minor porins is very limited. Previously, we have described a new OmpY porin in *Y. pseudotuberculosis* initially predicted from the genomic data. OmpY was recovered as a recombinant protein but we failed in isolating the native protein (Solo'veva et al. 2011). We found that the gene encoding this porin is also presents in *Y. ruckeri* genome. Both the functional role of OmpY and the conditions favoring its expression in *Yersinia* are still unknown.

**Figure 5:**
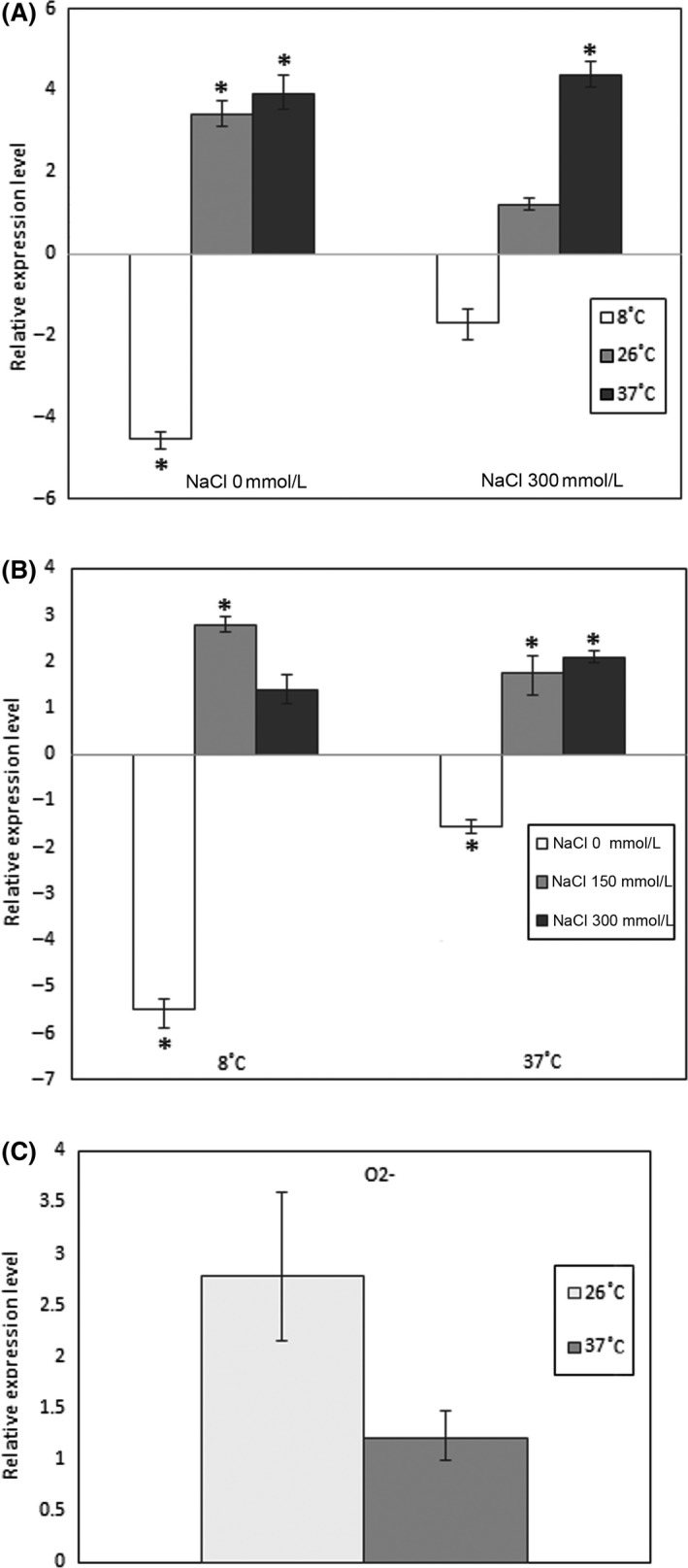
OmpY relative expression under different environmental conditions. (A) Effect of media osmolarity, 150 mmol/L NaCl condition was used as a reference. (B) Effect of cultivation temperature, 26°C condition was used as a reference. (C) Effect of oxygen availability, aerobic condition was used as a reference. Expression levels are reported as fold change compared to average expression levels of reference groups. Columns represent the mean of triplicate measurements in two independent experiments; error bars represent the standard deviations. Asterisks above bars indicate significant differences compared to the reference (*P* < 0.05).

It is evident from qRT‐PCR data (Fig. 5A) that the decreased osmolarity downregulates the *ompY* transcription (4.5‐fold) at 8°C and upregulates its transcription (3.4‐fold) at 26°C. Furthermore, increasing or decreasing of osmolarity led to the same effect, namely, to increased transcription (approximately fourfold) of *ompY* at high temperature. Lowering the temperature increased the *ompY* transcription by approximately threefold at 8°C (Fig. 5B), a temperature when a maximum reduction of *ompC* transcription was documented (Fig. 2). The transcription of the minor *ompY* porin gene increased insignificantly in response to anaerobiosis (Fig. 5C), whereas the transcription of major porins decreased (Fig. 4). It should be noted that *ompY* transcription behavior was very similar to the *ompF* one under most tested conditions. Because OmpY is supposed to present in small amounts in the outer membrane, the differences in the OMP profiles might be undetectable under tested conditions. It is also possible that we could not define the particular stress conditions necessary for production of considerable amounts of OmpY porin in *Y. ruckeri*.

Thus, several stress conditions may affect the *ompY* transcription that indicates its special role in maintaining the necessary level of porins in the bacterial cell.

## Conclusion

In summary, we report in this work that regulation of the porin balance in *Y. ruckeri*, in spite of some similarities, diverges from that system in *E. coli*. The changes in porin regulation can be adapted in *Y. ruckeri* in a species‐specific manner determined by its aquatic habitats. The study of a minor porin is of particular interest as it could provide the necessary level of porins at the special stress conditions.

## Conflict of Interest

None declared.

## Supporting information


**Figure S1.** SDS‐PAGE analysis of porin samples, reflecting effects of growth temperature and osmolarity.

**Figure S2.** SDS‐PAGE analysis of porin samples, reflecting effects of oxygen availability.
