# Supplementary figures and images for: Adaptive responses of outer membrane porin balance of Yersinia ruckeri under different incubation temperature, osmolarity, and oxygen availability
Source: Microbiologyopen. 2016 Apr 1;5(4):597–603. doi: 10.1002/mbo3.354 (PMC4985593; doi:10.1002/mbo3.354)

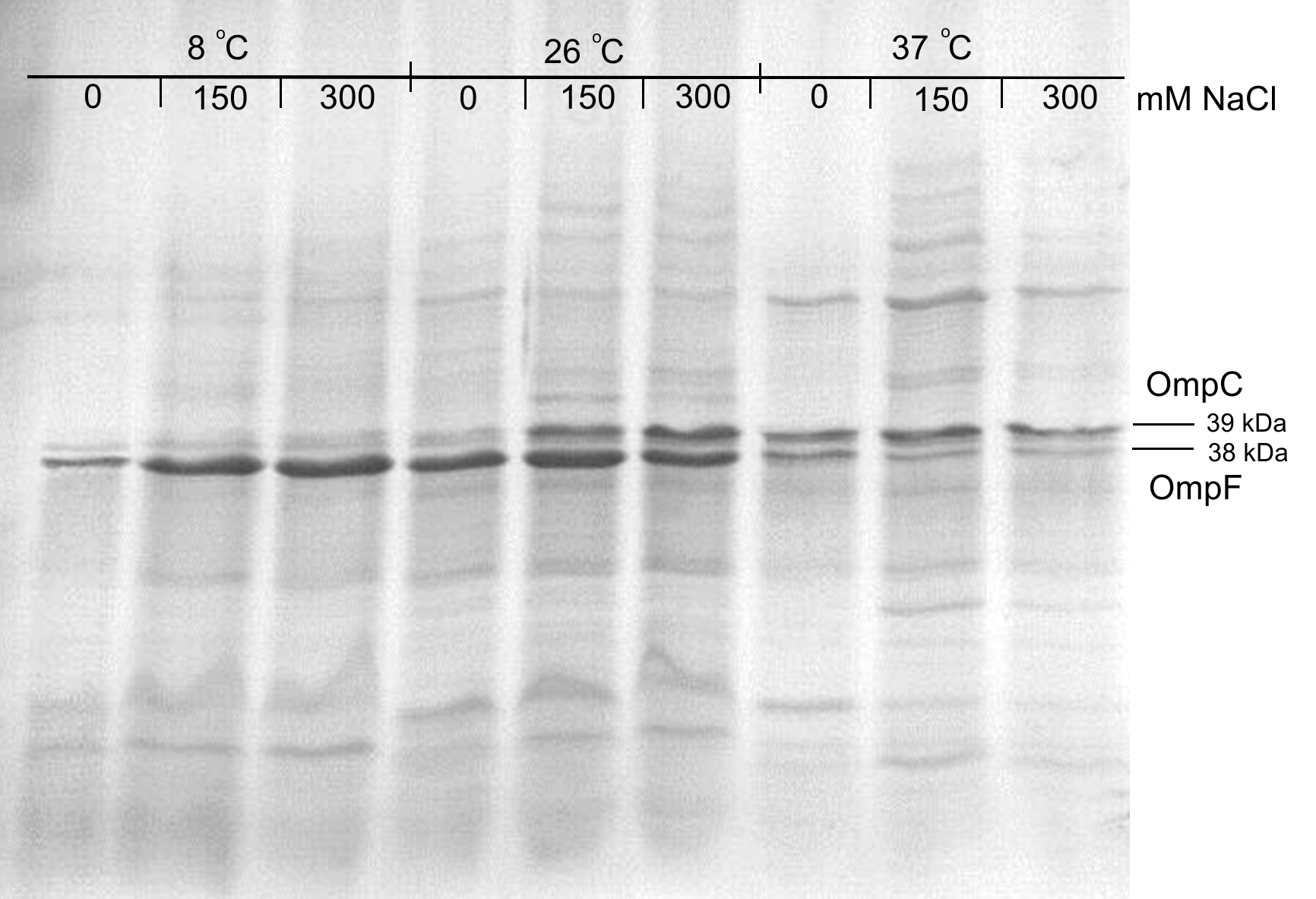

Supplement: Supplementary file 1 — Figure S1. SDS‐PAGE analysis of porin samples, reflecting effects of growth temperature and osmolarity. [file MBO3-5-597-s001.tif]

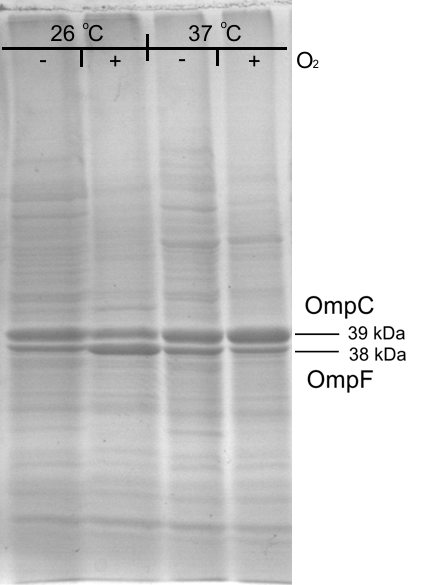

Supplement: Supplementary file 2 — Figure S2. SDS‐PAGE analysis of porin samples, reflecting effects of oxygen availability. [file MBO3-5-597-s002.tif]
